# Early physical rehabilitation vs standard care for intracerebral hemorrhage stroke: A protocol for systematic review and meta-analysis: Retraction

**DOI:** 10.1097/MD.0000000000030540

**Published:** 2022-09-02

The article, “Early physical rehabilitation vs standard care for intracerebral hemorrhage stroke: A protocol for systematic review and meta-analysis”,^[1]^ which published in Volume 100, Issue 3 of *Medicine* is being retracted. The authors requested a retraction due to errors in the research methods, including major deviations with the age of patients and outcome indicators, thus affecting the validity of the results. For the sake of scientific accuracy, all authors agreed to retract the article.
